# Temporal Evolution of Contrast Enhancement in Cranial Nerves: Insights from Heavily T2-Weighted 3D FLAIR Imaging

**DOI:** 10.3390/jimaging12100503

**Published:** 2026-10-12

**Authors:** Iichiro Osawa, Keita Nagawa, Hirokazu Shimizu, Taira Shiratori, Kaiji Inoue

**Affiliations:** Department of Radiology, Saitama Medical University Hospital, Saitama 350-0495, Japan; knagawa@saitama-med.ac.jp (K.N.); h_smz@saitama-med.ac.jp (H.S.); tori_taira@yahoo.co.jp (T.S.); kaiji@saitama-med.ac.jp (K.I.)

**Keywords:** oculomotor nerve, trigeminal nerve, facial nerve, cranial nerve, 3D FLAIR, MR imaging, contrast enhancement, gadolinium-based contrast agent

## Abstract

Background: Cranial nerve enhancement is traditionally assessed with T1-weighted imaging, but heavily T2-weighted 3D FLAIR (HT2-FLAIR) imaging offers greater sensitivity to subtle T1 shortening. This study aimed to assess contrast enhancement of the oculomotor, trigeminal, and facial nerves using HT2-FLAIR imaging. Methods: This retrospective repeated-measures cohort study included 33 patients. We subjectively assessed contrast enhancement of the cisternal segments of these three cranial nerves across precontrast, postcontrast, and 4 h delayed postcontrast HT2-FLAIR images and objectively evaluated serial changes in the signal intensity ratio (SIR)—the ratio of each structure’s signal intensity to that of the pons—for the oculomotor and trigeminal nerves, cerebrospinal fluid (CSF) spaces, and choroid plexus. Results: Subjectively, a high rate of enhancement (>90%) was observed bilaterally for all three nerves on 4 h delayed postcontrast images. Objectively, SIR of both the oculomotor and trigeminal nerves peaked on 4 h delayed postcontrast images, followed by postcontrast images, paralleling the pattern of CSF spaces and differing from that of choroid plexus. Conclusions: This study is the first to show that HT2-FLAIR imaging can visualize temporal enhancement of the cisternal segments of cranial nerves. Extravascular gadolinium may represent an alternative mechanism for normal cranial nerve enhancement.

## 1. Introduction

Contrast enhancement of cranial nerves on MR imaging generally has been assessed using T1-weighted (T1W) imaging. Normal cranial nerves usually do not enhance except for some nerve segments [1,2,3]. For example, the geniculate, tympanic, and mastoid segments of the normal facial nerve show contrast enhancement on T1W imaging, while the labyrinthine, canalicular, and cisternal segments do not enhance [1].

Abnormal cranial nerve enhancement on T1W imaging is clinically important because it can be the first or only imaging finding of an underlying disease process, including neoplastic conditions such as perineural tumor spread and schwannoma, and infectious, inflammatory, or granulomatous neuritis [4,5]. Because normal cranial nerve segments essentially lack enhancement on T1W imaging, any visible enhancement of these segments has traditionally been regarded as abnormal and has prompted further clinical work-up. Accurate differentiation between pathological and non-pathological enhancement therefore depends on a precise understanding of the enhancement behavior of each cranial nerve segment under conditions in which the nerve itself is not diseased.

Recently, conventional 3D FLAIR imaging has emerged as an alternative to evaluate contrast enhancement of cranial nerves [6]. Because 3D FLAIR-based sequences are sensitive to subtle T1-shortening effects of gadolinium [7], they can depict enhancement not visible on T1W imaging. Furthermore, a direct comparison of contrast-enhanced 3D FLAIR and contrast-enhanced 3D T1W imaging in patients with idiopathic facial palsy found that conventional T1W imaging showed reduced diagnostic specificity, attributable in part to enhancement of the normal facial nerve. In contrast, contrast-enhanced 3D FLAIR imaging achieved higher specificity in the same comparison. The authors concluded that 3D FLAIR imaging was superior to 3D T1W imaging in improving diagnostic performance in patients with idiopathic facial palsy [8].

Among the various 3D FLAIR-based sequences, heavily T2-weighted (T2W) 3D FLAIR (HT2-FLAIR) is distinguished by an increased sensitivity to subtle T1-shortening relative to the conventional 3D FLAIR technique [9,10,11,12,13]. This approach was used to detect subtle T1-shortening across a range of anatomical sites, including the inner ear [10,11], cerebrospinal fluid (CSF) spaces [12], and CSF leakage [9,13]. However, to the best of our knowledge, no study has fully examined cranial nerves on HT2-FLAIR imaging.

Because a fully healthy control group was not available in our retrospective dataset, we examined cranial nerves that were themselves free of clinical or radiological abnormality in patients imaged for suspected endolymphatic hydrops, a condition not known to directly affect these nerves. We hypothesized that HT2-FLAIR imaging, owing to its greater sensitivity to subtle T1 shortening, would identify contrast enhancement of the cisternal segments of these cranial nerves that is not detectable on conventional T1W imaging. We further hypothesized that, because gadolinium is known to distribute within CSF spaces after intravenous administration [14], a temporal enhancement pattern in these nerve segments could provide additional insight into the exchange of gadolinium and other CSF-borne substances between the CSF space and the cranial nerves. The purpose of our study is to assess the presence and temporal evolution of contrast enhancement of the oculomotor, trigeminal, and facial nerves using HT2-FLAIR imaging in this cohort.

## 2. Materials and Methods

### 2.1. Patients

This single-center, retrospective, repeated-measures study was approved by our Institutional Review Board and evaluated 36 consecutive patients with suspected endolymphatic hydrops imaged between March 2017 and July 2022. The inclusion criteria required the availability of the following MR imaging: (1) MR cisternography (MRC) and (2) HT2-FLAIR imaging obtained with and without a gadolinium-based contrast agent. All patients gave written informed consent for the MR imaging procedures, while the retrospective evaluation of clinical records was conducted under an institutionally approved opt-out policy. For patients under 18 years of age, written consent and opt-out approval were obtained from their parent or legal guardian. Candidates were excluded based on the following criteria: (1) insufficient image quality for interpretation; (2) a field of view (FOV) that did not cover the cranial nerves; (3) presence of clinical or radiological abnormalities involving the cranial nerves; and (4) radiological abnormalities in any other structures selected for measurement. Two board-certified radiologists (I.O. and K.N.) evaluated the cranial nerves and all other structures selected for measurement in every candidate for such abnormalities, and eligibility was determined by consensus between the two readers. Clinical abnormalities were identified from a review of each patient’s medical records. No formal sample-size calculation was performed. The sample size (*n* = 33) was determined by the number of consecutive patients meeting the eligibility criteria for our institution’s existing endolymphatic hydrops-imaging protocol, representing a retrospective convenience sample.

### 2.2. MR Imaging

All MR examinations were performed on a 3-Tesla system (MAGNETOM Skyra, Siemens, Erlangen, Germany) equipped with a 32-channel head coil. Using a protocol for evaluating endolymphatic hydrops [12], we acquired axial HT2-FLAIR and axial heavily T2W MRC images of the inner ear in 36 patients. One patient underwent precontrast, postcontrast, and 4 h delayed postcontrast fat-suppressed T1W imaging in the axial plane (TR 700 ms, TE 23 ms, a frequency-selective fat-suppression prepulse, and 0.3 mm slice thickness). HT2-FLAIR imaging was conducted using the following parameters: TR 9000 ms, TE 542 ms, inversion time 2250 ms, a frequency-selective fat suppression prepulse, variable flip-angle echo train with an average flip angle of 120°, echo train length 519, matrix size 384 × 384, FOV 166 mm × 196 mm, axial slices 104, 0.5 mm × 0.5 mm in-plane resolution at a slice thickness of 1.0 mm, bandwidth 434 Hz/pixel, an acceleration factor 2 using the GRAPPA parallel imaging technique, number of excitations 2, and acquisition time 7 min 17 s. Gadolinium-HP-DO3A (gadoteridol) was administered intravenously at a single dose of 0.1 mmol/kg (0.2 mL/kg). Postcontrast HT2-FLAIR imaging was performed 0.5 to 5 min after injection, and delayed postcontrast HT2-FLAIR imaging was obtained 4 h later.

MRC was acquired with the following parameters: TR 4400 ms, TE 542 ms, a frequency-selective fat suppression prepulse, variable flip-angle echo train with an average flip angle of 120°, echo train length 519, matrix size 384 × 384, FOV 166 mm × 196 mm, axial slices 104, 0.5 mm × 0.5 mm in-plane resolution at a slice thickness of 1.0 mm, bandwidth 434 Hz/pixel, an acceleration factor 2 using the GRAPPA parallel imaging technique, number of excitations 1.8, and acquisition time 3 min 15 s.

### 2.3. Imaging Analysis

Images were analyzed subjectively and objectively by the two radiologists who were blinded to clinical information. Images from the three time points were presented to each reader in a randomized order, and patient identity and time point were anonymized during image reading. All images were independently reviewed by the two radiologists, and discrepancies in a subjective analysis were resolved through discussion to reach the final consensus, while quantitative data obtained from an objective analysis by the two radiologists were presented as means and standard deviations (SDs).

#### 2.3.1. Subjective Analysis

For the subjective evaluation, overall contrast enhancement was visually assessed in the cisternal segments of the bilateral oculomotor, trigeminal, and facial nerves. Contrast enhancement of a nerve segment was defined as a higher signal intensity (SI) on postcontrast or 4 h delayed postcontrast HT2-FLAIR imaging compared to that on precontrast imaging. SI was compared between postcontrast and 4 h delayed postcontrast HT2-FLAIR imaging and classified into three categories: higher on postcontrast imaging, equal, and higher on 4 h delayed postcontrast imaging. Two board-certified radiologists, blinded to the patients’ clinical data, independently evaluated all images. Any discrepancies between the two radiologists were resolved through discussion to reach a consensus. Subsequently, symmetry of contrast enhancement between the left and right cranial nerves was evaluated on postcontrast and 4 h delayed postcontrast HT2-FLAIR imaging. We defined a difference as unilateral enhancement (one side enhanced and the other unenhanced), and no difference as bilateral enhancement or bilateral absence of enhancement.

#### 2.3.2. Objective Analysis

**Figure 1:**
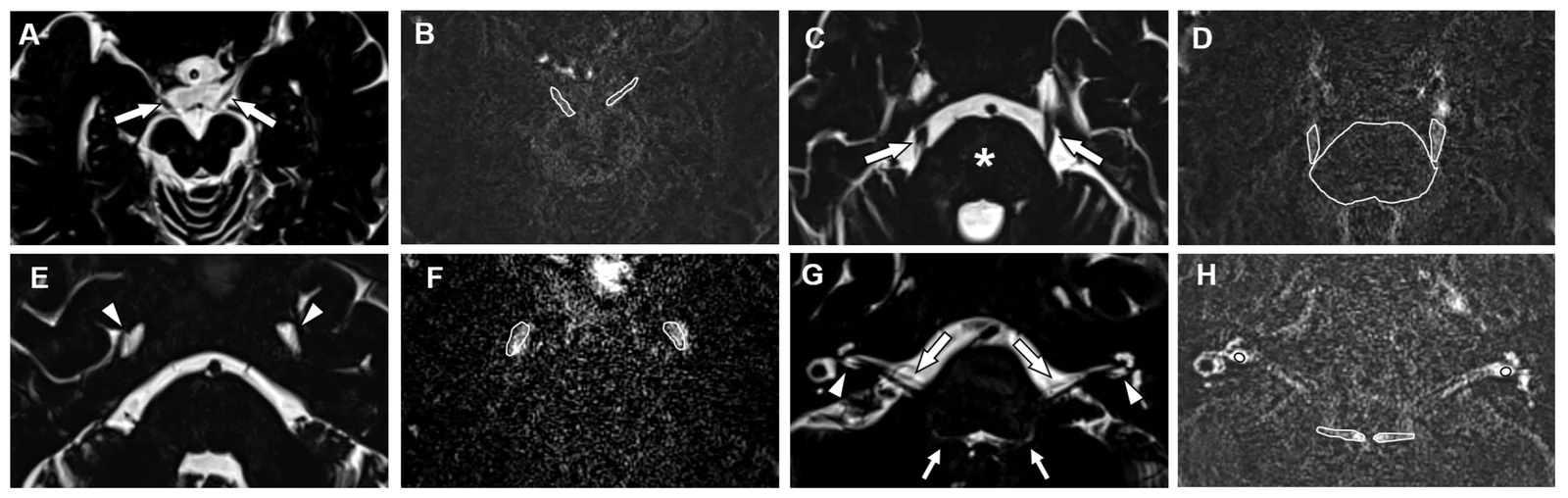
Region of interest (ROI) placements. (**A**) The oculomotor nerve (arrows) on MR cisternography (MRC). (**B**) Freehand ROI placed bilaterally in the oculomotor nerve on 4 h delayed postcontrast heavily T2-weighted 3D FLAIR (HT2-FLAIR) imaging. (**C**) The trigeminal nerve (arrows) and pons (asterisk) on MRC. (**D**) Freehand ROI placed in the trigeminal nerve bilaterally and the pons on 4 h delayed postcontrast HT2-FLAIR imaging. (**E**) Meckel’s cave (arrowheads) on MRC. (**F**) Freehand ROI placed in Meckel’s cave bilaterally on 4 h delayed postcontrast HT2-FLAIR imaging. (**G**) The facial nerve (arrows), fundus of the internal auditory canal (IAC) (arrowheads), and choroid plexus of the fourth ventricle (short arrows) on MRC. (**H**) Ovoid ROI and freehand ROI placed bilaterally in the fundus of the IAC and choroid plexus, respectively, on 4 h delayed postcontrast HT2-FLAIR imaging. ROI analysis is not performed on the facial nerve because of its small size.

An objective analysis was conducted by measuring the SI in predefined regions of interest (ROIs) on the workstation (Synapse VINCENT version 5.2, Fujifilm, Tokyo, Japan). Figure 1 illustrates examples of ROI placement. ROIs were manually placed on cranial nerves (oculomotor and trigeminal nerves), CSF spaces (Meckel’s cave and fundus of the internal auditory canal [IAC]), choroid plexus of the fourth ventricle, and brainstem (pons) on HT2-FLAIR imaging with reference to the corresponding MRC, and then the mean SI within each ROI was calculated at each time point. For cranial nerves, we drew freehand ROIs on the cisternal segment of bilateral nerves and calculated SI as the mean of the left and right sides. The mean size of the ROI on oculomotor nerves and trigeminal nerves was 15.2 mm^2^ (range 11.6–22.6 mm^2^) and 29.7 mm^2^ (range 17.3–44.3 mm^2^), respectively. Objective SI assessment of the facial nerve was not performed. The cisternal segment of the facial nerve is considerably smaller in caliber than the oculomotor and trigeminal nerves. A freehand ROI drawn on this segment is disproportionately affected by partial volume effects, and the resulting measurement is not sufficiently reliable. We therefore restricted objective analysis to the oculomotor and trigeminal nerves.

With regard to CSF spaces, freehand ROIs (mean 18.7 mm^2^, range 10.3–29.8 mm^2^) and ovoid ROIs (mean 3.5 mm^2^, range 2.0–6.0 mm^2^) were drawn bilaterally on the Meckel’s cave and fundus of IAC, respectively, and then SI was calculated as the mean of both sides. For the choroid plexus, ROI placement (mean 14.2 mm^2^, range 8.0–24.0 mm^2^) and SI were calculated in the same manner as for the Meckel’s cave. For the brainstem, a freehand ROI (mean 527 mm^2^, range 326–639 mm^2^) was placed over the entire pons at the level of the trigeminal nerve root.

Two radiologists measured the SI, and the SI ratio (SIR) for each patient was calculated using the following formula: SIR = SI of each structure (cranial nerves, CSF spaces, or choroid plexus)/SI of the pons. The mean SIR from the two observers was then calculated, and the final data were expressed as the means and SDs for all patients.

#### 2.3.3. Outcomes, Predictors, and Potential Confounders/Effect Modifiers

The primary outcomes were the subjective presence/absence of contrast enhancement and the objective SIR of each structure. The principal predictor of interest was imaging time point (precontrast, postcontrast, 4 h delayed postcontrast). Potential confounders considered included renal function (estimated glomerular filtration rate), patient age, and sex (Supplementary Table S1). Laterality and severity of endolymphatic hydrops were considered as potential effect modifiers (Supplementary Table S1), given the possibility that disease-related alterations in inner-ear and CSF homeostasis could influence the magnitude or pattern of nerve/CSF enhancement independently of the imaging time point itself.

### 2.4. Statistical Analysis

Repeated measurements of SIR across three time points were analyzed using the Friedman test, followed by post hoc pairwise comparisons using the Wilcoxon signed-rank test with the Holm correction for multiple comparisons. Gwet’s AC1 and the intraclass correlation coefficient (ICC) (2, 1) were used as indices of interreader reliability. Gwet’s AC1 was used instead of assessing Cohen’s kappa because this method overcomes the limitation of kappa being sensitive to trait prevalence and marginal probability [15]. ICC (2, 1) was estimated with a two-way random effects model of absolute agreement for a single observation. AC1 and ICC values were interpreted according to the following classifications by Landis and Koch [16]: <0, indicating no agreement; 0–0.20, slight agreement; 0.21–0.40, fair agreement; 0.41–0.60, moderate agreement; 0.61–0.80, substantial agreement; and 0.81–1, almost perfect agreement. Details for SIR comparisons are provided in Supplementary Table S2. To assess the stability of the pons reference region used for SIR normalization, raw (non-normalized) pons SI values obtained by both readers were compared across the three time points (Supplementary Table S3), using the Friedman test, followed by the Wilcoxon signed-rank test and Holm correction for multiple comparisons. Interreader agreement for pons SI at each time point was quantified using the ICC (2, 1). Because this was a single-arm, repeated-measures design with within-subject comparisons across time points, no adjustment for confounding was performed. Time-invariant patient-level confounders are controlled for by this design. No data were missing for either the subjective or objective analyses. Complete visual assessments and ROI measurements were obtained for all 33 patients at all three time points. No sensitivity analyses were performed in this study. All statistical analyses were performed using SPSS software (version 27.0; IBM Corp., Armonk, NY, USA) and R (version 4.4.5; R Foundation for Statistical Computing, Vienna, Austria). Two-tailed *p*-values < 0.05 were considered statistically significant.

## 3. Results

Thirty-six patients underwent MRC and HT2-FLAIR imaging with and without gadolinium-based contrast agent. Of the 36 patients, 33 with available images met the inclusion criteria. Three patients were excluded because of no visualization of bilateral oculomotor nerves due to bilateral chronic subdural hematoma associated with CSF leaks (*n* = 1), vestibular schwannoma within the fundus of the right IAC (*n* = 1), or left trigeminal neuralgia caused by neurovascular compression (*n* = 1). The patient cohort included 14 males and 19 females, with an average age of 54 years (range 17–80 years). Detailed demographic and clinical characteristics of the 33 included patients are summarized in Supplementary Table S1.

### 3.1. Subjective Analysis

**Figure 2:**
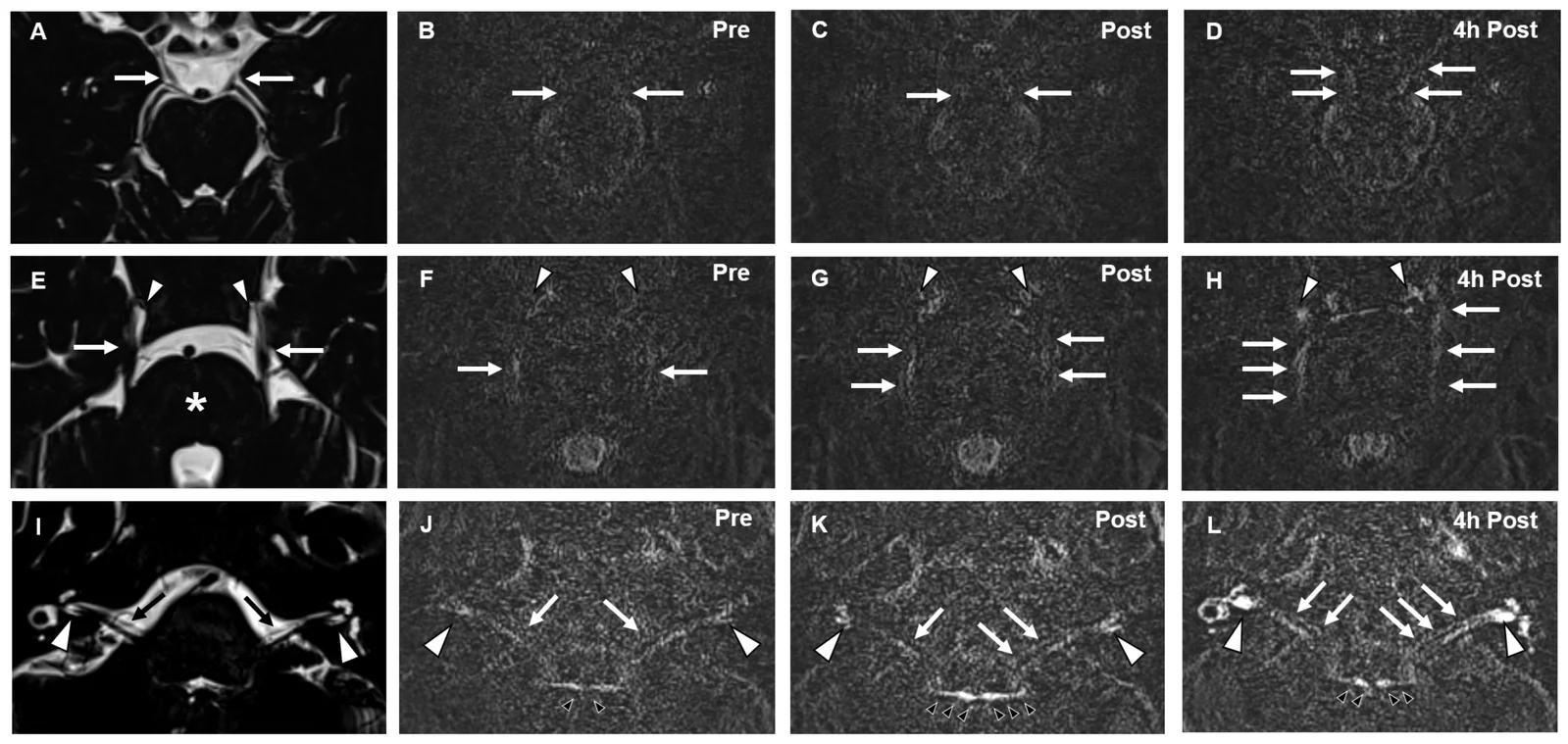
Serial changes in contrast enhancement of cranial nerves. (**A**) The cisternal segment of the oculomotor nerve (arrows) on MR cisternography (MRC). (**B**–**D**) The cisternal segment of bilateral oculomotor nerves (arrows) on precontrast (**B**), postcontrast (**C**), and 4 h delayed postcontrast (**D**) heavily T2-weighted 3D FLAIR (HT2-FLAIR) imaging. This segment shows contrast enhancement on both postcontrast and 4 h delayed postcontrast imaging, which is stronger on 4 h delayed postcontrast imaging than on postcontrast imaging. (**E**) The cisternal segment of the trigeminal nerve (arrows), Meckel’s cave (arrowheads), and pons (asterisk) on MRC. (**F**–**H**) The cisternal segment of the bilateral trigeminal nerves (arrows), Meckel’s caves (arrowheads), and pons on precontrast (**F**), postcontrast (**G**), and 4 h delayed postcontrast (**H**) HT2-FLAIR imaging. The nerves and Meckel’s caves show contrast enhancement on both postcontrast and 4 h delayed postcontrast imaging, which is stronger on 4 h delayed postcontrast imaging than on postcontrast imaging. The pons exhibits no enhancement on either postcontrast or 4 h delayed postcontrast imaging. (**I**) The cisternal segment of the facial nerve (arrows) and fundus of the internal auditory canal (IAC) (arrowheads) on MRC. (**J**–**L**) The cisternal segment of bilateral facial nerves (arrows) and fundus of bilateral IACs (arrowheads) on precontrast (**J**), postcontrast (**K**), and 4 h delayed postcontrast (**L**) HT2-FLAIR imaging. The facial nerve and fundus of the IAC show enhancement patterns parallel to those of the other cranial nerves and Meckel’s cave, respectively. The choroid plexus of the fourth ventricle shows stronger contrast enhancement on postcontrast imaging than on 4 h delayed postcontrast imaging (black arrowheads).

**Figure 3:**
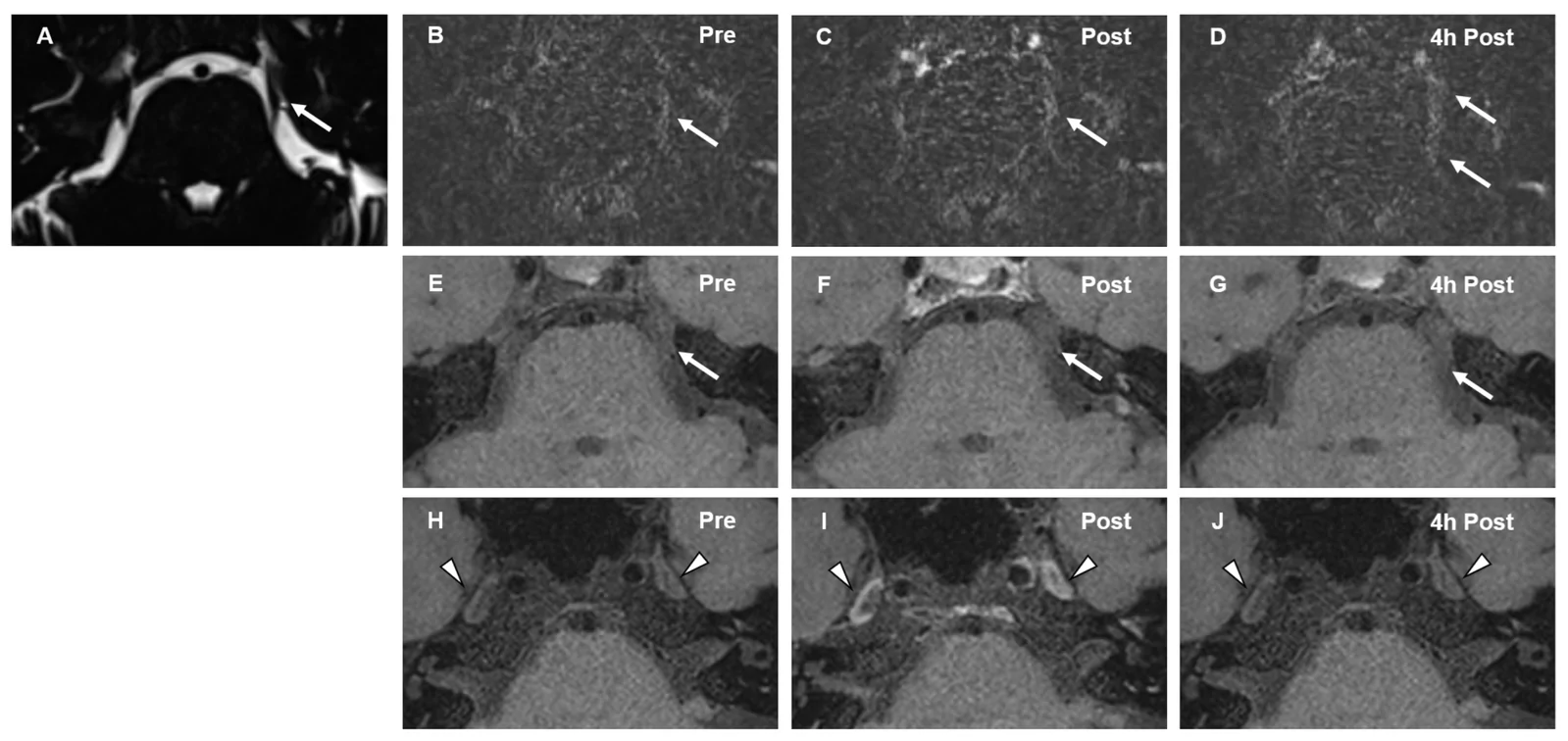
The cisternal segment of the trigeminal nerve. (**A**) The cisternal segment of left trigeminal nerve (arrow) on MR cisternography (MRC). (**B**–**D**) The cisternal segment of left trigeminal nerve (arrows) on precontrast (**B**), postcontrast (**C**), and 4 h delayed postcontrast (**D**) heavily T2-weighted 3D FLAIR (HT2-FLAIR) imaging. This segment is enhanced on both postcontrast and 4 h delayed postcontrast imaging. Contrast enhancement is stronger on 4 h delayed postcontrast imaging than on postcontrast imaging. (**E**–**G**) The cisternal segment of the left trigeminal nerve (arrows) on precontrast (**E**), postcontrast (**F**), and 4 h delayed postcontrast (**G**) T1-weighted (T1W) imaging. This segment shows no enhancement on either postcontrast or 4 h delayed postcontrast imaging. Also, contrast enhancement is not observed in either the oculomotor or facial nerves. (**H**–**J**) Perineural vascular plexus (arrowheads) on precontrast (**H**), postcontrast (**I**), and 4 h delayed postcontrast (**J**) T1W imaging. Contrast enhancement of this plexus is observed on both postcontrast and 4 h delayed postcontrast T1W imaging, which is stronger on postcontrast imaging than on 4 h delayed postcontrast imaging.

Table 1 shows a summary of results for contrast enhancement of the three cranial nerves. Figure 2 demonstrates serial changes of contrast enhancement of the three cranial nerves. All of the three cranial nerves showed contrast enhancement on postcontrast and 4 h delayed postcontrast imaging. On 4 h delayed postcontrast imaging, enhancement was observed bilaterally in all patients for the oculomotor and trigeminal nerves (100%) and in most patients for the facial nerve (94–97%). These rates were higher than those observed on postcontrast imaging (58–82% for the oculomotor and trigeminal nerves, 3–12% for the facial nerve). In addition, contrast enhancement was observed more frequently on 4 h delayed postcontrast imaging than postcontrast imaging. Interreader reliability was substantial to almost perfect for contrast enhancement of cranial nerves (postcontrast imaging; AC1 = 0.71–0.94, 4 h delayed postcontrast imaging; AC1 = 0.90–1.00). In a single illustrative case, HT2-FLAIR imaging depicted enhancement of the cisternal segment of the left trigeminal nerve on both postcontrast and 4 h delayed postcontrast images (Figure 3), whereas the same nerve showed no enhancement on either postcontrast or 4 h delayed postcontrast T1W imaging in this patient. Contrast enhancement was similarly not observed in the oculomotor or facial nerves on T1W imaging in this patient. This comparison was performed in a single patient and represents a proof-of-principle observation rather than a cohort-level finding.

Table 2 shows differences in SI of cranial nerves between postcontrast and 4 h delayed postcontrast imaging, and differences in contrast enhancement between left and right nerves. SI on 4 h delayed postcontrast imaging was equal to or higher than that on postcontrast imaging in all of the three cranial nerves. Interreader reliability was almost perfect (AC1 = 0.86–1.00). When comparing contrast enhancement between left and right nerves, the “no difference” category was observed more frequently than the “difference” category in all of the three cranial nerves.

### 3.2. Objective Analysis

**Figure 4:**
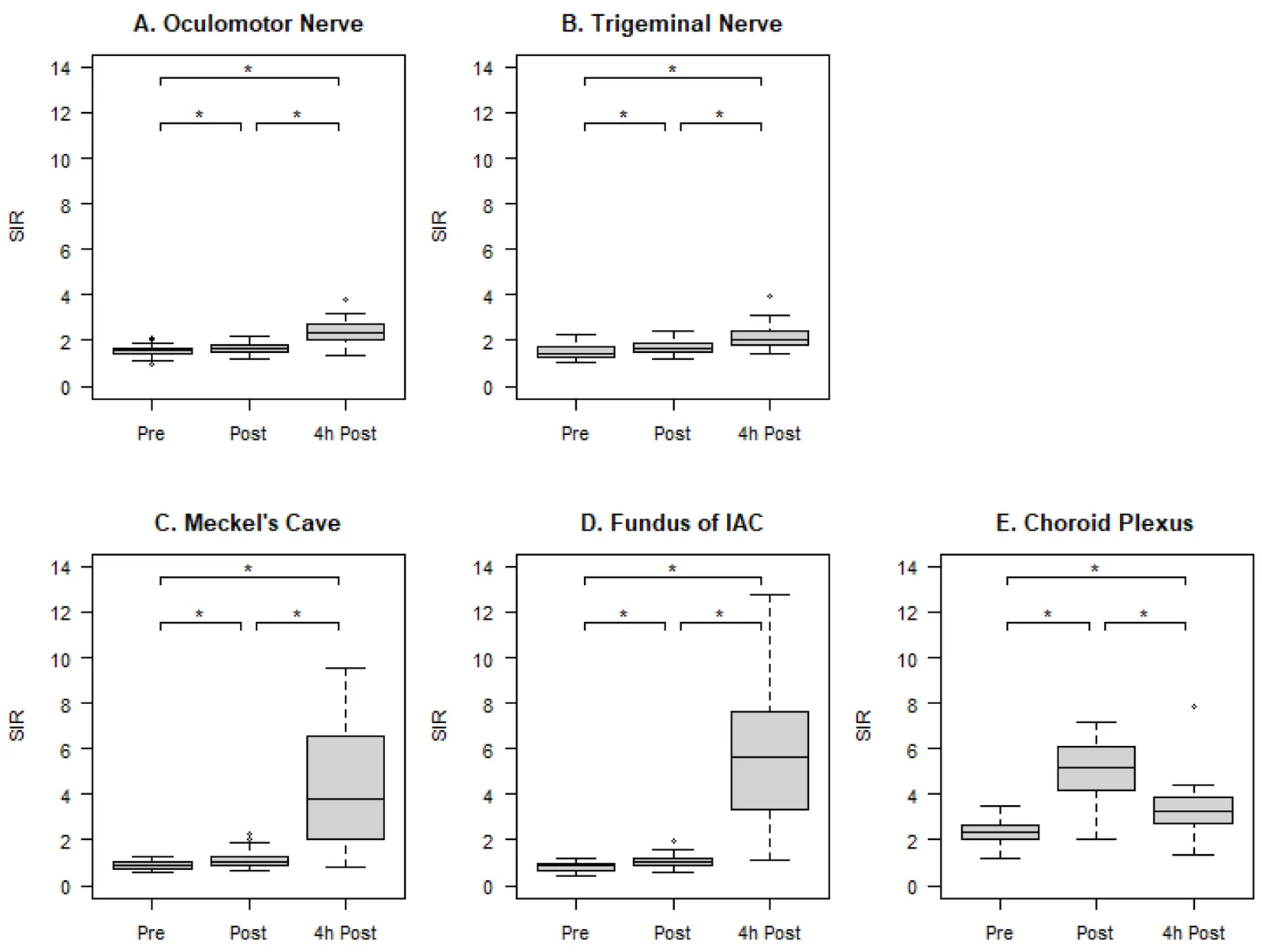
Box-and-whisker plots representing signal intensity ratio (SIR) trends over time for the cranial nerves, cerebrospinal fluid (CSF) spaces, and choroid plexus on heavily T2-weighted 3D FLAIR (HT2-FLAIR) imaging. (**A**,**B**) SIR plots for cranial nerves (oculomotor and trigeminal nerves). These nerves show statistically significantly higher enhancement on postcontrast and 4 h delayed postcontrast imaging compared with precontrast imaging. 4 h delayed postcontrast imaging exhibits higher SIR than postcontrast imaging. (**C**,**D**) SIR plots for CSF spaces (Meckel’s cave and fundus of internal auditory canal [IAC]). These spaces demonstrate enhancement patterns parallel to those of the cranial nerves. (**E**) SIR plots for the choroid plexus. This plexus shows statistically significantly higher enhancement on postcontrast and 4 h delayed postcontrast imaging compared with precontrast imaging. Contrast enhancement of the plexus is statistically significantly stronger on postcontrast imaging than 4 h delayed postcontrast imaging. Note: Asterisks (*) indicate statistical significance (*p* < 0.05).

Table 3 demonstrates SIR of cranial nerves and other structures, and Figure 4 shows chronological comparisons of SIR. SIR in both of the oculomotor and trigeminal nerves was statistically significantly higher on postcontrast (oculomotor nerve; *p* < 0.001, trigeminal nerve; *p* < 0.0001) and 4 h delayed postcontrast (both *p* < 0.0001) HT2-FLAIR imaging than precontrast imaging. In these nerves, 4 h delayed postcontrast imaging exhibited higher SIR than postcontrast imaging (both *p* < 0.0001).

Meckel’s cave and fundus of IAC showed enhancement patterns parallel to those of the cranial nerves; SIR in both of the CSF spaces was statistically significantly higher on postcontrast (both *p* < 0.0001) and 4 h delayed postcontrast (both *p* < 0.0001) HT2-FLAIR imaging than precontrast imaging. SIR in the choroid plexus was statistically significantly higher on postcontrast (*p* < 0.0001) and 4 h delayed postcontrast (*p* < 0.0001) imaging compared with precontrast imaging. Moreover, SIR was higher on postcontrast imaging than on 4 h delayed postcontrast imaging (*p* < 0.0001). Interreader reliability was substantial to almost perfect (ICC (2, 1) = 0.64–0.99). Details including effect sizes for these comparisons are provided in Supplementary Table S2.

Mean (SD) raw pons SI was 8.92 (1.17) on precontrast, 9.12 (1.26) on postcontrast, and 9.02 (1.33) on 4 h delayed postcontrast imaging. Post hoc pairwise comparisons with Holm correction showed a small but statistically significant increase between precontrast and postcontrast imaging (*p* = 0.015), whereas the precontrast vs. 4 h delayed postcontrast comparison (*p* = 0.243) and the postcontrast vs. 4 h delayed postcontrast comparison (*p* = 0.442) were not significant. Interreader agreement for pons SI was almost perfect at all time points (ICC = 1.00, 1.00, and 0.99, respectively). The statistical details, including effect sizes, are provided in Supplementary Table S3.

## 4. Discussion

All of the three cranial nerves, including the oculomotor, trigeminal, and facial nerves, showed contrast enhancement on HT2-FLAIR imaging, which was stronger on 4 h delayed postcontrast imaging than on postcontrast imaging. The timing and magnitude of the SI within the cisternal segment of the cranial nerves followed a similar trend to that of CSF spaces.

Relative to T1W imaging, FLAIR imaging is more sensitive to subtle T1-shortening arising from low gadolinium concentrations or elevated protein content. The 3D variant further improves anatomical delineation over its 2D counterpart because of higher spatial resolution and the ability to generate high-quality multiplanar and 3D reconstructions [17], while also reducing CSF flow-related artifacts [17]. Within the family of 3D FLAIR imaging, HT2-FLAIR imaging affords an even greater degree of sensitivity to subtle T1 shortening than the conventional technique [9], and has previously been applied to structures such as the inner ear [10,11], CSF spaces [12], and CSF leaks [9,13]. No prior report has described enhancement of the normal cisternal cranial nerve segments on T1W imaging. To the best of our knowledge, the present study is the first to demonstrate time-dependent enhancement of the cisternal segment of the cranial nerves using HT2-FLAIR imaging.

The oculomotor nerve arises at the midbrain and then enters the interpeduncular cistern. Subsequently, the nerve runs anteroinferiorly in the prepontine cistern, and travels through the cavernous sinus into the orbit. Contrast enhancement of the cisternal segment of the oculomotor nerve is always abnormal on T1W imaging [2]. The oculomotor nerve can enhance in various pathological conditions, including inflammatory and neoplastic processes. A previous report evaluated the SI pattern of the normal oculomotor nerve on contrast-enhanced 3D FLAIR imaging [18]. However, because that study did not include precontrast 3D FLAIR imaging, contrast enhancement of the normal oculomotor nerve could not be assessed.

The trigeminal nerve has both sensory and motor roots arising from the pons, and travels to the oromaxillofacial regions. The sensory root enters Meckel’s cave via the prepontine cistern, forming the trigeminal ganglion, and is divided into three main branches: ophthalmic (V1), maxillary (V2), and mandibular (V3). The motor root bypasses the trigeminal ganglion and unites with the sensory root to become the mandibular nerve. The trigeminal ganglion, which lies in Meckel’s cave, is surrounded by a perineural vascular plexus. The trigeminal nerve can be divided into five anatomical segments: brainstem, cisternal, Meckel’s cave and cavernous sinus, skull base, and extracranial segments [19]. Along the cisternal segment, central myelin (oligodendrocyte) extending from the brainstem meets peripheral myelin (Schwann cell). The portion of the nerve along which the central myelin transitions to peripheral myelin is referred to as the transition zone, also known as the Obersteiner–Redlich zone. The trigeminal ganglion or its maxillary or mandibular branches enhance infrequently on T1W imaging, which is supported by their avascular appearance in cadaveric specimens [3].

The facial nerve has motor, sensory, and parasympathetic nerve fibers. The facial nerve runs through the IAC and courses through the facial canal in the temporal bone. The facial nerve can be divided into six anatomical segments: cisternal, internal auditory canalicular, labyrinthine, geniculate, tympanic, and mastoid. The geniculate, tympanic, and mastoid segments of the normal facial nerve show contrast enhancement on T1W imaging [1]. Gebarski et al. suggested that these segments have a rich arteriovenous plexus within their perineurium and epineurium, and that contrast enhancement may occur as the contrast agent pools within this plexus [1]. On the other hand, Tabuchi et al. examined the vascular permeability of human cranial nerves, including the facial nerve, using intravenously administered fluorescein [20]. Fluorescence was observed in the external nerve sheath and slightly in the endoneurium of the normal facial nerve. These findings suggest that the vascular barrier in human peripheral nerves is incomplete. These researchers hypothesized that the gadolinium enhancement is caused by the vascular permeability of the nerve.

In contrast, the labyrinthine, canalicular, and cisternal segments of the normal facial nerve do not enhance on T1W imaging [1]. With the development of MR imaging such as improved spatial resolution and higher static magnetization, several reports have shown that contrast enhancement is observed in the labyrinthine and canalicular segments of the normal facial nerve on T1W imaging [21,22,23]. However, to the best of our knowledge, there have been no reports describing normal enhancement of the cisternal segment of the facial nerve.

The question then arises: how does the cisternal segment of normal cranial nerves enhance? Although various explanations such as the glymphatic system [24] and the intramural periarterial drainage (IPAD) pathway [25] have been postulated, the precise mechanism remains unknown. We can propose two hypotheses to explain this finding: (1) intravascular enhancement and (2) extravascular enhancement. With regard to the first hypothesis, normal enhancement of the facial nerve reflects pooling of contrast agent within the arteriovenous plexus located in the perineurium and epineurium, as mentioned earlier [1]. However, the cisternal segment of the cranial nerves lacks a perineurium and epineurium, so the arteriovenous plexus may not exist in this segment. While the choroid plexus shows marked early enhancement as a highly vascularized structure lacking a blood–brain barrier, the cranial nerves in the present study showed an enhancement pattern parallel to that of the CSF spaces. These findings suggest that nerve enhancement is predominantly extravascular, distinguishing it from the typical vascular and extravascular enhancement seen in the choroid plexus.

With regard to the second hypothesis, there are three possible routes for gadolinium to access the cranial nerves: (1) blood vessels, (2) CSF, and (3) brain parenchyma. Regarding the first possible route, gadolinium leakage may occur in the peripheral portion of the cranial nerves from blood vessels to the perineural space. Nakao et al. examined vascular permeability in the rabbit cranial nerves, including the oculomotor, trigeminal, and facial nerves after intravenous injection of sodium fluorescein [26]. Fluorescence was identified in the distal nerves as follows: the intracavernous segment of the oculomotor nerve, the distal IAC segment of the facial nerve, and the trigeminal ganglion. In in vivo MR study after intravenous gadolinium administration, the trigeminal nerve was enhanced in the fluorescence-positive area. Histological examination revealed intense fluorescence in the ganglia and external nerve sheaths of the cranial nerves. A slight fluorescence was also observed in endoneurial connective tissue but not in the nerve fibers. These researchers suggest that the physiological gadolinium enhancement of human cranial nerves may correlate with vascular permeability. It should be noted that the fluorescein-based evidence discussed above, from both human [20] and rabbit [26] studies, cannot directly establish the mechanism of gadolinium enhancement in human cranial nerves. Fluorescein and gadolinium chelates differ substantially in molecular size and physicochemical properties. Nakao et al. also conducted their study in rabbits rather than humans. These findings should therefore be regarded as indirect, supportive evidence rather than direct proof of the proposed mechanism. In addition, the trigeminal ganglion lacks a barrier system, which allows substances such as neuropeptides released in the ganglion to enter the circulation [27]. These findings raise a question: can gadolinium move from the peripheral portion to the central portion of the cranial nerves? Some substances can be transferred along the olfactory and/or trigeminal nerve pathways after intranasal administration [28,29]. These nerve pathways can involve the pathways along the perineural (endoneurium, perineurium, or epineurium) and perivascular spaces. Thus, in the present study, gadolinium transport may occur from the peripheral to the central portion of the cranial nerves.

Regarding the second possible route, previous studies have revealed that substances injected into CSF drain along cranial nerves [28]. In addition, gadolinium has been shown to be distributed in the CSF spaces after intravenous administration [14]. The pia mater covers the surface of the brain and spinal cord and can be fenestrated in both the brain [30] and spinal cord [31]. The pia mater also covers the central portion of cranial nerves [32]. If gadolinium may move from the subarachnoid space to cranial nerves, although the exact mechanism is unclear, pial fenestration may allow for communication between the subarachnoid space and the subpial space of cranial nerves, as well as for gadolinium transport to the subpial space. Moreover, gadolinium may partially enter cranial nerves via the glymphatic pathway.

Regarding the third possible route, Kanda et al. revealed gadolinium deposition in the brain after its intravenous administration [33]. Although the precise mechanism remains unknown, a recent paper proposed a gadolinium inflow route via the CSF and directly from the blood [34]. And then, gadolinium may move from the brainstem to cranial nerves by some unknown mechanism. In the present study, however, we exclude this scenario because the normal brainstem usually does not show contrast enhancement.

These findings have several important clinical implications. First, the observation that the cisternal segments of cranial nerves can demonstrate contrast enhancement on HT2-FLAIR imaging indicates that such enhancement should not be automatically interpreted as pathological. Because HT2-FLAIR imaging is substantially more sensitive to subtle T1-shortening effects than T1W imaging, enhancement detected on HT2-FLAIR imaging alone may lead to overestimation of inflammatory, infectious, or neoplastic processes if interpreted in isolation. Therefore, cranial nerve enhancement on HT2-FLAIR imaging should be evaluated in conjunction with T1W imaging and clinical findings to avoid misdiagnosis. In addition, pathological conditions affecting cranial nerves may alter the enhancement pattern on HT2-FLAIR imaging, potentially revealing abnormalities that are not detectable on contrast-enhanced T1W imaging. Because of the high sensitivity of HT2-FLAIR imaging to low concentrations of gadolinium, this technique may provide complementary diagnostic information in disorders such as neuritis, perineural tumor spread, or demyelinating disease. The ability of HT2-FLAIR imaging to detect subtle changes in the perineural or subarachnoid environment suggests that it could serve as a valuable adjunct in the evaluation of cranial nerve pathology. Finally, the presence of delayed enhancement in cranial nerves raises the possibility that HT2-FLAIR imaging reflects physiological interactions between cranial nerves and surrounding CSF-related pathways, including glymphatic or perivascular transport. Although speculative, this sensitivity may eventually contribute to the development of imaging biomarkers for subtle alterations in nerve–CSF dynamics in various neurological disorders. Further studies are warranted to clarify how these enhancement patterns can be leveraged in clinical decision-making.

There were several limitations that need to be addressed. First, the current study was retrospective in nature with its small sample size. Second, we did not evaluate other cranial nerves such as the acoustic nerve. Further research is warranted to assess contrast enhancement of such cranial nerves. Third, we evaluated patients with suspected endolymphatic hydrops. Although these patients did not show neurological symptoms associated with the cranial nerves, they were not completely healthy subjects. Endolymphatic hydrops can involve altered inner-ear fluid and barrier homeostasis, and we cannot exclude that CSF-gadolinium kinetics in this cohort differ from those in a healthy population. The parallel enhancement kinetics observed between cranial nerves and CSF spaces may in part reflect disease-related change rather than a physiological process. We did not perform a subgroup analysis comparing patients with and without endolymphatic hydrops. Therefore, a possible contribution of hydrops to the observed enhancement cannot be excluded. Future studies should evaluate these findings in a prospective, multi-institutional cohort including healthy controls, and should also include subgroup analyses stratified by hydrops severity. Fourth, this study has an observational design. It cannot establish causality or determine which of the three proposed gadolinium transport pathways produced the observed enhancement. Each route discussed above remains hypothetical. Dedicated mechanistic or pharmacokinetic studies will be needed to confirm them. Fifth, freehand ROIs were used to measure SI in the cisternal nerves. Because these nerves are small in caliber, partial volume contamination from adjacent CSF cannot be excluded. The parallel time course between the nerves and CSF spaces observed in this study may reflect this contamination rather than a true signal change confined to the nerve itself. We did not perform a separate analysis to exclude this possibility. This measurement-validity concern should be considered when interpreting the reported nerve SIR values. Sixth, a ROI-based assessment of the facial nerve was not performed. The cisternal segment of the facial nerve is considerably smaller in caliber than that of the oculomotor and trigeminal nerves, making it more susceptible to partial-volume contamination, and a reliable ROI could not be placed on this structure. Enhancement of the facial nerve was therefore evaluated only through subjective visual analysis. Seventh, the comparison between HT2-FLAIR and T1W imaging was performed in a single patient. This comparison should be regarded as a proof-of-principle observation, and the apparent greater sensitivity of HT2-FLAIR relative to T1W imaging has not been replicated at the cohort level. Eighth, raw pons SI, used as the denominator for the SIR, increased by about 2.5% between precontrast and postcontrast imaging, and this change could, in principle, affect the calculated SIR. However, the pons SI increased rather than decreased at the postcontrast time point. A rise in the denominator would be expected to slightly underestimate, not inflate, the SIR. This early change in pons SI is therefore unlikely to account for the increase in SIR observed for the cranial nerves and CSF spaces on postcontrast imaging. Pons SI remained stable between postcontrast and 4 h delayed postcontrast imaging. This finding indicates that the further increase in SIR observed at the 4 h delayed time point cannot be attributed to a change in the pons reference signal. Taken together, these results indicate that the observed increase in SIR across the three imaging time points is unlikely to be explained by changes in the pons reference signal. Ninth, no formal sample size calculation was performed. The cohort represented a retrospective convenience sample drawn from an existing endolymphatic hydrops imaging protocol. The precision of our estimates should be interpreted with this limitation in mind. Tenth, postcontrast imaging was obtained approximately 0.5 to 5 min after contrast administration. This interval varied among patients and may have introduced some inconsistency into the postcontrast measurements. Finally, data were collected at a few time points. More time points need to be examined in future studies.

## 5. Conclusions

In this retrospective, single-center cohort of patients with suspected endolymphatic hydrops, the cisternal segments of the oculomotor, trigeminal, and facial nerves showed contrast enhancement on HT2-FLAIR imaging, which was more pronounced on 4 h delayed postcontrast than on postcontrast images. This enhancement pattern paralleled that of adjacent CSF spaces and differed from the earlier, more pronounced enhancement observed in the choroid plexus, suggesting that extravascular gadolinium distribution may contribute to cranial nerve enhancement in addition to intravascular pooling. Because our cohort lacked a fully healthy control group, these findings should be regarded as hypothesis-generating rather than definitive evidence of a universal physiological phenomenon. Future studies should aim to (1) evaluate these findings in a prospective, multi-institutional cohort that includes healthy controls without suspected endolymphatic hydrops or other inner-ear pathology; (2) extend the ROI-based analysis to additional cranial nerves, including the facial nerve, using acquisition protocols that minimize partial-volume contamination from adjacent CSF and other structures; and (3) add a greater number of standardized postcontrast time points to better characterize the temporal kinetics of enhancement. Such studies would help determine whether this enhancement pattern on HT2-FLAIR imaging can be reliably distinguished from pathological enhancement in clinical practice. Furthermore, such research may provide new insight into substance transport along the cranial nerves.

## Figures and Tables

**Table 1:** Contrast enhancement of cranial nerves on subjective visual analysis.

	Contrast Enhancement				AC1 (95% CI)			
	Post		4 h Post		Post		4 h Post	
	Rt	Lt	Rt	Lt	Rt	Lt	Rt	Lt
Oculomotor nerve	27 (82%)	24 (73%)	33 (100%)	33 (100%)	0.81 (0.61–1.01)	0.81 (0.61–1.01)	1.00 (1.00–1.00)	1.00 (1.00–1.00)
Trigeminal nerve	19 (58%)	21 (64%)	33 (100%)	33 (100%)	0.71 (0.47–0.96)	0.78 (0.56–1.00)	1.00 (1.00–1.00)	0.97 (0.90–1.03)
Facial nerve	1 (3%)	4 (12%)	31 (94%)	32 (97%)	0.94 (0.84–1.03)	0.82 (0.65–0.99)	0.90 (0.79–1.02)	0.97 (0.90–1.03)

CI—confidence interval, Post—postcontrast heavily T2-weighted 3D FLAIR imaging, 4 h Post—4 h delayed postcontrast heavily T2-weighted 3D FLAIR imaging.

**Table 2:** Differences in overall SI and contrast enhancement of cranial nerves on subjective visual analysis.

	Differences in Overall SI Between Post vs. 4 h Post				Differences in Contrast Enhancement Between Left vs. Right Nerves					
					Post			4 h Post		
	Post Higher	Equal	4 h Post Higher	AC1 (95% CI)	Difference	No Difference	AC1 (95% CI)	Difference	No Difference	AC1 (95% CI)
Oculomotor nerve	0 (0%)	2 (6%)	31 (94%)	0.98 (0.94–1.01)	0	33	1.00 (1.00–1.00)	0	33	1.00 (1.00–1.00)
Trigeminal nerve	0 (0%)	0 (0%)	33 (100%)	0.99 (0.98–1.01)	1	32	0.97 (0.90–1.03)	0	33	1.00 (1.00–1.00)
Facial nerve	0 (0%)	0 (0%)	33 (100%)	0.97 (0.90–1.03)	2	31	0.86 (0.72–1.01)	0	33	0.94 (0.84–1.03)

SI*—*signal intensity, Post—postcontrast heavily T2-weighted 3D FLAIR imaging, 4 h Post—4 h delayed postcontrast heavily T2-weighted 3D FLAIR imaging, CI—confidence interval.

**Table 3:** SIR of cranial nerves and other structures on HT2-FLAIR imaging.

	SIR (SD)			ICC (95% CI)		
	Pre	Post	4 h Post	Pre	Post	4 h Post
Oculomotor nerve	1.54 (0.24)	1.67 (0.24) *	2.41 (0.50) *#	0.93 (0.83–0.97)	0.92 (0.84–0.96)	0.92 (0.85–0.96)
Trigeminal nerve	1.50 (0.33)	1.67 (0.30) *	2.14 (0.51) *#	0.97 (0.93–0.98)	0.96 (0.92–0.98)	0.97 (0.94–0.98)
Meckel’s cave	0.89 (0.17)	1.14 (0.38) *	4.23 (2.56) *#	0.94 (0.89–0.97)	0.92 (0.84–0.96)	0.98 (0.96–0.99)
Fundus of IAC	0.83 (0.20)	1.07 (0.28) *	5.50 (2.65) *#	0.64 (0.39–0.81)	0.67 (0.42–0.82)	0.99 (0.97–0.99)
Choroid plexus	2.29 (0.53)	5.00 (1.29) *†	3.28 (1.11) *	0.95 (0.91–0.98)	0.96 (0.90–0.98)	0.96 (0.92–0.98)

SIR—signal intensity ratio, SD—standard deviation, CI—confidence interval, HT2-FLAIR*—*heavily T2-weighted 3D FLAIR, Pre—precontrast heavily T2-weighted 3D FLAIR imaging, Post—postcontrast heavily T2-weighted 3D FLAIR imaging, 4 h Post—4 h delayed postcontrast heavily T2-weighted 3D FLAIR imaging, ICC—intraclass correlation coefficient, IAC*—*internal auditory canal. Asterisk (*), number sign (#), and dagger sign (†) indicate statistically significantly higher values (*p* < 0.05) versus precontrast, postcontrast, and 4 h delayed postcontrast imaging, respectively.

## Data Availability

The datasets generated and analyzed during the current study are not publicly available due to their containing information that could compromise patient privacy and confidentiality but are available from the corresponding author on reasonable request.

## Supplementary Materials

The following supporting information can be downloaded at: https://www.mdpi.com/article/10.3390/jimaging12100503/s1, Table S1: Demographic and clinical characteristics of the study cohort; Table S2: Effect size of signal intensity ratio (SIR) comparisons; Table S3: Signal intensity (SI) of pons; STROBE Statement checklist. References [35,36] are cited in the Supplementary Materials.

## Abbreviations

The following abbreviations are used in this manuscript:

T1W	T1-weighted
T2W	T2-weighted
HT2-FLAIR	Heavily T2-weighted 3D FLAIR
CSF	Cerebrospinal fluid
MRC	MR cisternography
FOV	Field of view
SD	Standard deviation
SI	Signal intensity
ROI	Region of interest
IAC	Internal auditory canal
SIR	Signal intensity ratio
ICC	Intraclass correlation coefficient
IPAD	Intramural periarterial drainage
