# Supplementary material for: Temporal Evolution of Contrast Enhancement in Cranial Nerves: Insights from Heavily T2-Weighted 3D FLAIR Imaging
Source: J Imaging. 2026 Oct 12;12(10):503. doi: 10.3390/jimaging12100503 (PMC13705865; doi:10.3390/jimaging12100503)
Supplement: Supplementary file 1 [file jimaging-12-00503-s001.zip › STROBE Statement Checklist.pdf]

Journal of
Imaging

MDPI

Supplementary Materials

Temporal Evolution of Contrast Enhancement in Cranial Nerves: Insights from Heavily T2-Weighted 3D FLAIR Imaging

Iichiro Osawa *, Keita Nagawa, Hirokazu Shimizu, Taira Shiratori and Kaiji Inoue

Department of Radiology, Saitama Medical University Hospital, Saitama 350-0495, Japan; knagawa@saitama-med.ac.jp (K.N.); h_smz@saitama-med.ac.jp (H.S.); tori_taira@yahoo.co.jp (T.S.); kaiji@saitama-med.ac.jp (K.I.)

* Correspondence: oyabun@saitama-med.ac.jp; Tel.: +81-49-276-1265

STROBE Statement Checklist

This checklist was modified from the STROBE Statement—Checklist of items that should be included in reports of cohort studies.

Item No.	Recommendation	Reported in manuscript (section)	Page / Line no.
	**Title and Abstract**		
1a	Indicate the study's design with a commonly used term in the title or the abstract.	Abstract, Materials and Methods	p.1, p.2
1b	Provide in the abstract an informative and balanced summary of what was done and what was found.	Abstract	p.1
	**Introduction**		
2	Explain the scientific background and rationale for the investigation being reported.	Introduction	p.1–2
3	State specific objectives, including any prespecified hypotheses.	Introduction	p.2
	**Methods**		
4	Present key elements of study design early in the paper.	Materials and Methods	p.2
5	Describe the setting, locations, and relevant dates, including periods of recruitment, exposure, follow-up, and data collection.	Materials and Methods	p.2
6a	Give the eligibility criteria, and the sources and methods of selection of participants.	Materials and Methods	p.2–3
6b	For matched studies, give matching criteria and number of exposed/unexposed.	Not applicable.	—

Item No.	Recommendation	Reported in manuscript (section)	Page / Line no.
7	Clearly define all outcomes, exposures, predictors, potential confounders, and effect modifiers.	Materials and Methods	p.4
8	For each variable of interest, give sources of data and details of methods of assessment. Describe comparability of assessment methods.	Materials and Methods	p.4
9	Describe any efforts to address potential sources of bias.	Materials and Methods	p.3
10	Explain how the study size was arrived at.	Materials and Methods	p.3
11	Explain how quantitative variables were handled in the analyses; describe groupings chosen and why.	Materials and Methods	p.3–4
12a	Describe all statistical methods, including those used to control for confounding.	Materials and Methods	p.4–5
12b	Describe any methods used to examine subgroups and interactions.	Not performed; disclosed in Discussion/Limitations.	p.13–14
12c	Explain how missing data were addressed.	Materials and Methods	p.5
12d	Cohort study—explain how loss to follow-up was addressed.	Not applicable.	—
12e	Describe any sensitivity analyses.	Materials and Methods	p.5
		**Results**	
13a/b/c	Report numbers of individuals at each stage; give reasons for non-participation; consider a flow diagram.	Results, first paragraph: 36 imaged → 33 included → 3 excluded (reasons given).	p.5
14a	Give characteristics of study participants and information on exposures/potential confounders.	Results, first paragraph: age/sex; reference to Supplementary Table S1.	p.5
14b	Indicate number of participants with missing data for each variable of interest.	Covered by the Item 12c statement (no missing data).	p.5
14c	Cohort study—summarise follow-up time.	Materials and Methods, §2.2: postcontrast 0.5–5 min; delayed at 4h.	p.3
15	Report numbers of outcome events or summary measures over time.	Results, §3.1: delayed-enhancement rates stated as percentages.	p.5
16a	Give unadjusted (and adjusted) estimates and their precision (e.g., 95% CI).	Results, Supplementary Table S2 and S3.	p.5–9

https://doi.org/10.3390/jimaging12100503

Item No.	Recommendation	Reported in manuscript (section)	Page / Line no.
16b	Report category boundaries when continuous variables were categorized.	Materials and Methods, §2.3.1: 3-category classification defined.	p.3–4
16c	Consider translating relative risk into absolute risk.	Not applicable.	—
17	Report other analyses done (subgroups, interactions, sensitivity analyses).	Results, §3.2: raw pons SI results reported. No subgroup or sensitivity analyses performed (see 12b, 12e).	p.9
		**Discussion**	
18	Summarise key results with reference to study objectives.	Discussion, opening paragraph	p.13
19	Discuss limitations, taking into account sources of potential bias or imprecision.	Discussion, Limitations paragraph	p.13–14
20	Give a cautious overall interpretation of results considering objectives, limitations, multiplicity of analyses, and other evidence.	Discussion	p.11–14
21	Discuss the generalisability (external validity) of the study results.	Discussion (Limitations) and Conclusions	p.13–15
		**Other Information**	
22	Give the source of funding and the role of the funders.	Funding statement: "This research received no external funding."	p.15
