# Supplementary material for: Temporal Evolution of Contrast Enhancement in Cranial Nerves: Insights from Heavily T2-Weighted 3D FLAIR Imaging
Source: J Imaging. 2026 Oct 12;12(10):503. doi: 10.3390/jimaging12100503 (PMC13705865; doi:10.3390/jimaging12100503)
Supplement: Supplementary file 1 [file jimaging-12-00503-s001.zip › Supplementary Tables.pdf]

Journal of Imaging

MDPI

Supplementary Materials

Temporal Evolution of Contrast Enhancement in Cranial Nerves: Insights from Heavily T2-Weighted 3D FLAIR Imaging

Iichiro Osawa *, Keita Nagawa, Hirokazu Shimizu, Taira Shiratori and Kaiji Inoue

Department of Radiology, Saitama Medical University Hospital, Saitama 350-0495, Japan; knagawa@saitama-med.ac.jp (K.N.); h_smz@saitama-med.ac.jp (H.S.); tori_taira@yahoo.co.jp (T.S.); kaiji@saitama-med.ac.jp (K.I.)

* Correspondence: oyabun@saitama-med.ac.jp; Tel.: +81-49-276-1265

Table S1. Demographic and clinical characteristics of the study cohort.

Patient No.	Age (years)	Sex	Symptoms				Time from onset to MR imaging	Endolymphatic hydrops				eGFR (mL/min/1.73 m^2)
			Hearing loss	Tinnitus	Ear fullness	Vertigo		Cochlea		Vestibule		
								R	L	R	L	
1	44	F	1	1	1	1	9 months	1 (M)	1 (S)	0	0	67.3
2	24	M	1	1	1	0	13 days	1 (M)	1 (S)	1 (M)	1 (M)	118.8
3	17	F	0	1	0	0	15 days	1 (S)	1 (S)	0	0	122.4
4	49	M	0	1	0	1	1 year 1 month	1 (S)	1 (S)	0	0	84.5
5	33	F	1	1	0	1	3 months	1 (S)	1 (S)	0	0	82.9
6	62	F	1	1	0	0	13 days	0	0	0	0	72.7
7	69	M	1	0	0	0	10 months	1 (S)	0	1 (M)	1 (S)	93.8
8	49	M	0	1	0	0	11 years	0	0	0	0	105.3
9	80	M	0	0	0	1	10 months	1 (M)	1 (S)	0	0	50.7
10	30	F	0	0	0	1	10 months	1 (S)	1 (S)	1 (S)	1 (S)	120.6
11	48	M	1	1	1	1	12 years	1 (S)	1 (S)	0	1 (S)	82.7
12	44	M	1	1	1	1	8 months	1 (S)	1 (M)	NA	0	76.3
13	61	F	0	0	1	1	7 years	1 (S)	1 (M)	0	1 (M)	55.3
14	70	F	0	1	0	1	1 year 11 months	0	1 (S)	0	1 (M)	72.7
15	71	F	1	0	1	1	1 year 6 months	0	0	0	0	64.3
16	56	F	1	1	0	1	1 year	0	1 (M)	0	1 (M)	72.3
17	49	F	1	0	0	1	3 years 3 months	0	0	1 (M)	1 (M)	66.2
18	59	F	1	1	1	1	11 years 9 months	1 (S)	0	1 (S)	1 (M)	70.1
19	69	F	1	0	0	1	1 year 10 months	0	0	0	0	62.3
20	61	F	1	1	1	1	8 years 11 months	0	1 (S)	0	1 (S)	96.2
21	67	F	0	0	0	1	7 months	0	0	1 (M)	1 (M)	75.0
22	63	F	1	1	1	0	3 years 11 months	0	0	1 (M)	1 (M)	47.0
23	49	M	0	0	0	1	3 years 7 months	0	0	0	0	88.3
24	30	M	0	0	0	1	6 months	0	0	0	1 (M)	102.2
25	73	M	0	0	0	1	1 year	0	0	0	0	59.7
26	69	M	1	1	0	1	19 days	NA	0	NA	0	90.7
27	53	M	1	1	1	0	4 months	1 (M)	0	0	1 (M)	54.3
28	56	F	1	0	0	1	1 year 11 months	1 (S)	1 (S)	0	1 (S)	90.4
29	54	F	0	0	0	1	5 years 1 month	0	0	0	0	63.4
30	52	M	0	0	0	1	2 years 3 months	0	0	1 (M)	1 (M)	56.5

31	60	M	1	0	0	1	8 years 11 months	0	0	1 (M)	1 (M)	68.6
32	56	F	0	0	0	1	1 year	NA	NA	NA	NA	94.7
33	62	F	1	0	1	0	4 months	0	0	0	0	33.2

eGFR—estimated glomerular filtration rate, CSF—cerebrospinal fluid, R—right, L—left, 1—positive, 0—negative, NA—not available, M—mild, S—significant.

eGFR was calculated using the three-variable Japanese equation for patients aged 18 years and older: eGFR (mL/min/1.73 m^2) = 194 × serum creatinine$^{-1.094}$ × age$^{-0.287}$ (× 0.739 if female). For patients younger than 18 years, eGFR was calculated using the bedside Schwartz equation: eGFR (mL/min/1.73 m^2) = 0.413 × height (cm) / serum creatinine (mg/dL).

We semi-quantitatively assessed endolymphatic hydrops of the cochlea and vestibule according to the criteria proposed by Nakashima et al. [35]. The grading system of cochlear endolymphatic hydrops was as follows: no = no bulging of the cochlear duct or no visualization of the cochlear duct, mild = the cochlear duct was bulging toward the scala vestibuli without exceeding the perilymphatic space of the scala vestibuli, significant = the cochlear duct exceeded the perilymphatic space of the scala vestibuli. Endolymphatic hydrops of the vestibule was graded as follows: no = less than 33% of the vestibule was occupied by the endolymph, mild = 33 to 50% of the vestibule was occupied by the endolymph, significant = more than 50% of the vestibule was occupied by the endolymph. Endolymphatic hydrops was independently evaluated by the two radiologists, and discrepancies in this evaluation were resolved through discussion to reach the final consensus.

Table S2. Effect size of signal intensity ratio (SIR) comparisons.

**Friedman test for SIR of cranial nerves and other structures**						
	n	SIR (SD)			Kendall's W (effect size)	Interpretation
		Pre	Post	4h Post		
Oculomotor nerve	33	1.54 (0.24)	1.67 (0.24)	2.41 (0.50)	0.82	Large
Trigeminal nerve	33	1.50 (0.33)	1.67 (0.30)	2.14 (0.51)	0.65	Large
Meckel’s cave	33	0.89 (0.17)	1.14 (0.38)	4.23 (2.56)	0.89	Large
Fundus of IAC	33	0.83 (0.20)	1.07 (0.28)	5.50 (2.65)	0.85	Large
Choroid plexus	33	2.29 (0.53)	5.00 (1.29)	3.28 (1.11)	0.91	Large

**Wilcoxon signed-rank test with Holm correction for SIR of cranial nerves and other structures**			
	Comparison	r (effect size)	Interpretation
Oculomotor nerve	Pre vs. Post	0.63	Large
Oculomotor nerve	Post vs. 4h Post	1.00	Large
Oculomotor nerve	Pre vs. 4h Post	1.00	Large
Trigeminal nerve	Pre vs. Post	0.75	Large
Trigeminal nerve	Post vs. 4h Post	0.93	Large
Trigeminal nerve	Pre vs. 4h Post	0.99	Large
Meckel’s cave	Pre vs. Post	0.89	Large
Meckel’s cave	Post vs. 4h Post	1.00	Large
Meckel’s cave	Pre vs. 4h Post	0.99	Large
Fundus of IAC	Pre vs. Post	0.86	Large

https://doi.org/10.3390/jimaging12100503

Fundus of IAC	Post vs. 4h Post	1.00	Large
Fundus of IAC	Pre vs. 4h Post	1.00	Large
Choroid plexus	Pre vs. Post	1.00	Large
Choroid plexus	Post vs. 4h Post	0.97	Large
Choroid plexus	Pre vs. 4h Post	0.98	Large

SIR—signal intensity ratio, SD—standard deviation, Pre precontrast heavily T2-weighted 3D FLAIR imaging, Post—postcontrast heavily T2-weighted 3D FLAIR imaging, 4h Post—4 h delayed postcontrast heavily T2-weighted 3D FLAIR imaging, IAC—internal auditory canal.

Effect-size magnitude was graded as negligible (< 0.1), small (0.1 –< 0.3), moderate (0.3 –< 0.5), or large (≥ 0.5), following Tomczak and Tomczak [36].

Table S3. Signal intensity (SI) of pons.

**Friedman test for SI of pons**									
	n	SI (SD)			ICC (95% CI)			Kendall's W (effect size)	Interpretation
		Pre	Post	4h Post	Pre	Post	4h Post		
Pons	33	8.92 (1.17)	9.12 (1.26)	9.02 (1.33)	1.00 (0.99–1.00)	1.00 (1.00–1.00)	0.99 (0.98–1.00)	0.09	Negligible

**Wilcoxon signed-rank test with Holm correction for SI of pons**				
	Comparison	Holm-adjusted *p*	r (effect size)	Interpretation
Pons	Pre vs. Post	0.015 *	0.58	Large
Pons	Post vs. 4h Post	0.442	0.32	Moderate
Pons	Pre vs. 4h Post	0.243	−0.15	Small

SI—signal intensity, SD—standard deviation, ICC—intraclass correlation coefficient (two-way random-effects, absolute agreement, single measures model), CI—confidence interval, Pre precontrast heavily T2-weighted 3D FLAIR imaging, Post postcontrast heavily T2-weighted 3D FLAIR imaging, 4h Post—4 h delayed postcontrast heavily T2-weighted 3D FLAIR imaging. Asterisk (*) indicates statistically significantly higher values ($p < 0.05$).

Mean SI within each ROI (region of interest) was recorded independently by the two radiologists, and their values were averaged for each patient. Finally, cohort-level results are reported as means and SDs.

Effect-size magnitude was graded as negligible (< 0.1), small (0.1 –< 0.3), moderate (0.3 –< 0.5), or large (≥ 0.5), following Tomczak and Tomczak [36].

Supplemental References

35. Nakashima, T.; Naganawa, S.; Pyykko, I.; Gibson, W.P.; Sone, M.; Nakata, S.; Teranishi, M. Grading of endolymphatic hydrops using magnetic resonance imaging. *Acta Otolaryngol Suppl* **2009**, *560*, 5-8.

36. Tomczak, M.; Tomczak, E. The need to report effect size estimates revisited. An overview of some recommended measures of effect size. *Trends Sport Sci.* 2014, 1(21), 19–25.

https://doi.org/10.3390/jimaging12100503
